# Role of HSP90 in Cancer

**DOI:** 10.3390/ijms221910317

**Published:** 2021-09-25

**Authors:** Bereket Birbo, Elechi E. Madu, Chikezie O. Madu, Aayush Jain, Yi Lu

**Affiliations:** 1 Massachusetts Institute of Technology, Cambridge, MA 02139, USA; bereketb@mit.edu; 2 Departments of Biological Sciences, University of Memphis, Memphis, TN 38152, USA; eemadu14@gmail.com (E.E.M.); comadu@memphis.edu (C.O.M.); ajain2@memphis.edu (A.J.); 3 Health Science Center, Department of Pathology and Laboratory Medicine, University of Tennessee, Memphis, TN 38163, USA

**Keywords:** HSP90, molecular chaperones, GRP94, TRAP1, targeted therapy

## Abstract

HSP90 is a vital chaperone protein conserved across all organisms. As a chaperone protein, it correctly folds client proteins. Structurally, this protein is a dimer with monomer subunits that consist of three main conserved domains known as the N-terminal domain, middle domain, and the C-terminal domain. Multiple isoforms of HSP90 exist, and these isoforms share high homology. These isoforms are present both within the cell and outside the cell. Isoforms HSP90*α* and HSP90*β* are present in the cytoplasm; TRAP1 is present in the mitochondria; and GRP94 is present in the endoplasmic reticulum and is likely secreted due to post-translational modifications (PTM). HSP90 is also secreted into an extracellular environment via an exosome pathway that differs from the classic secretion pathway. Various co-chaperones are necessary for HSP90 to function. Elevated levels of HSP90 have been observed in patients with cancer. Despite this observation, the possible role of HSP90 in cancer was overlooked because the chaperone was also present in extreme amounts in normal cells and was vital to normal cell function, as observed when the drastic adverse effects resulting from gene knockout inhibited the production of this protein. Differences between normal HSP90 and HSP90 of the tumor phenotype have been better understood and have aided in making the chaperone protein a target for cancer drugs. One difference is in the conformation: HSP90 of the tumor phenotype is more susceptible to inhibitors. Since overexpression of HSP90 is a factor in tumorigenesis, HSP90 inhibitors have been studied to combat the adverse effects of HSP90 overexpression. Monotherapies using HSP90 inhibitors have shown some success; however, combination therapies have shown better results and are thus being studied for a more effective cancer treatment.

## 1. Introduction

Heat-shock response was discovered in the 1960s by Italian scientist Ferruccio Ritossa who observed a characteristic puffing in the chromosomes of fruit flies that were unintentionally left in a high-heat environment [1]. Ritossa attributed the puffing of the chromosomes to the increased expression of specific proteins resulting from respective gene activation in response to heat stress [2]. Heat-shock proteins (HSP) were then discovered and became an important area of study because they are ubiquitous, found in all organisms, and function as chaperones that aid in repairing and correctly folding client proteins [3]. They also facilitate the intracellular transport of proteins within the cytosol, endoplasmic reticulum, and mitochondria [4]. In doing so, they protect cells from the adverse effects of heat and other factors [3,5].

The interaction of the heat-shock factor (HSF1) with heat-shock elements (HSEs) in the promoter regions of the heat-shock protein (HSP) gene triggers the transcription of HSP genes [6]. Although the HSP induction by heat stress occurs at varying temperatures among different organisms, in mammals, it occurs at fever temperatures [3,7].

Heat-shock proteins are classified based on the molecular mass of their monomer, ranging from 10 to greater than 100 kDa, their structure, and their function [2,6]. The primary heat-shock protein families are HSP100, 90, 70, 60, and the small HSP (sHSP) [6]. The focus of this review is HSP90. HSP90 refers to a subset of heat-shock proteins which have a molecular mass of 90 kDa. Within the HSP90 family, the various members are present in different cellular compartments. HSP90α and HSP90β are in the cytoplasm, GRP94 (94-kDa glucose-regulated protein) is present in the endoplasmic reticulum, and TRAP-1 (tumor necrosis factor receptor-associated protein 1) exists in the mitochondria [2].

HSP90 is an ATP-dependent molecular chaperone regulating the late-stage maturation, activation, and stability of various client proteins [8]. HSP90 proteins play a significant role in essential cellular processes and regulatory pathways such as apoptosis, cell cycle control, cell signaling, cell viability, protein folding, and degradation as they interact with client proteins and co-chaperones. Their prominent role lies in proteostasis, cell differentiation, and development [2]. Moreover, they play a role in the induction of adaptive immunity by associating with antigenic peptides and delivering them to antigen-presenting cells, thereby activating antigen-presenting cells as well as dendritic cells [2,9].

This review focuses on the link between the overexpression of HSP90 and cancer. A link between the overexpression of HSP90 and disease conditions, such as various types of cancer, viral infection, inflammation, and neurodegenerative diseases, has been observed, suggesting that HSP90 may contribute to cancer progression [10,11,12]. Understanding the link between the overexpression of HSP90 and cancer is crucial to finding better cancer treatments by exploiting the differences between normal and tumor cells in terms of HSP90 mRNA and protein induction, protein activation, and the number of post-translational modification (PTM) sites. Many of the HSP90 client proteins are involved in signal transduction and other vital pathways that are especially important in malignancy [8]. Knowing that the overexpression of HSP90 has a possible role in the development of cancer, researchers are studying the uses of HSP90 inhibitors to curb the overexpression of HSP90 and thereby treat cancer.

HSP90 inhibitors have only recently been used in clinical applications due to having been overlooked. One reason for having been overlooked is that HSP90 inhibitors would target HSP90, which is abundant in normal cells and vital for normal cellular processes. Regardless of the pleiotropic effects of HSP90, HSP90 inhibitors have become increasingly used in clinical trials [13].

## 2. Structure of HSP90

HSP90 is a dimer comprised of monomers with an overall structure consisting of three main conserved domains known as the N-terminal domain (NTD), C-terminal domain (CTD), and middle domain (MD) [10,14,15,16,17]. These domains are connected by linkers that allow the domains to rearrange as HSP90 undergoes conformational changes [18]. In eukaryotes, there exists a charged linker domain that connects the N-terminal and middle domains [19]. This charged linker domain varies in length and amino acid sequence composition [20].

Each of these domains takes on a specific function, and they are necessary for avoiding the accumulation of damaged or misfolded proteins, especially in aging [21]. The NTD is the binding site for ATP and is thus called the nucleotide-binding site [2]. Hence, the NTD is needed for HSP90 ATPase activity, necessary for the chaperone cycle and binding of client proteins to the HSP90 chaperone [22,23]. The CTD plays a significant role in protein dimerization. It has two main sites: one for calmodulin binding and another for homodimerization of HSP90 [24,25]. The CTD also has a nucleotide-binding site that opens when the NTD is occupied, thereby acting as an allosteric regulator of the N-terminal ATPase activity [26]. The CTD contains special motifs, MEEVD, or KDEL, which differ based on the HSP90 isoform and its location within the cell—either in the cytoplasm or the ER [2]. The MD functions in the binding of substrates. The MD also regulates HSP90 ATPase activity, as seen when the substrate binds to the MD, causing an increase in ATPase activity [27,28]. Although known to be present in only eukaryotic HSP90, the charged linker domain is essential for the function, interaction, and flexibility of the HSP90 chaperone [19].

Dimerization is necessary for the proper function of HSP90. Specifically, mammalian HSP90 is a phosphorylated dimer with monomers, each containing 2–3 covalently-bound phosphate molecules. In addition, despite having an ATP binding site in the NTD, HSP90 has not only ATPase activity but also GTPase activity [2].

**Figure 1:**
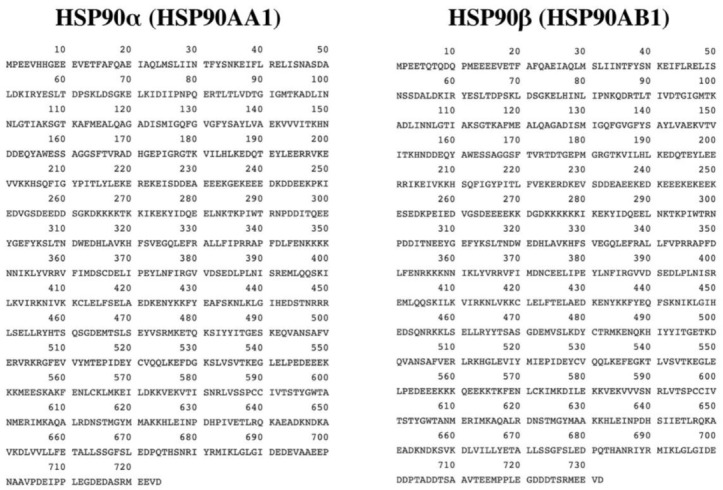
The amino acid sequences of isoforms HSP90α (HSP90AA1) and HSP90β (HSP90AB1) [31].

In mammals, there are two main isoforms of HSP90, which may result from duplicated genes [16,29]. These two isoforms are the inducible form HSP90α and the constitutive form HSP90β. They share 85% homology and are both found in the cytoplasm [30]. Despite being isoforms, HSP90α and HSP90β have some variation in specific sections of their protein sequences, as shown in Figure 1. HSP90*α* consists of 732 amino acids, while HSP90β consists of 724 amino acids. In addition, HSP90α tends to dimerize more frequently when compared to HSP90β. Although there are some differences between the two isoforms, HSP90 is often used to refer to both isoforms HSP90α and HSP90β, since they are similar in structure and function [2]. The similarities between these isoforms allow most of the client proteins to bind to either of these isoforms.

Another isoform of HSP90 is GRP94 (glucose-regulated protein 94). It shares 50% homology with cytoplasmic HSP90 and is found in the endoplasmic reticulum [29]. Because it is the most abundant glycoprotein present in the endoplasmic reticulum, it is also called endoplasmin [32,33]. It is similar to cytoplasmic isoforms of HSP90 in the way it binds and hydrolyzes ATP. Although it is like the cytoplasmic isoforms, being the major calcium-binding protein in the ER and having specific limited protein clients distinguish GRP94 from cytoplasmic HSP90 [34]. GRP94 exists in three conformations: extended, less extended, and closed conformations. The extended conformation allows for more access to client proteins and nucleotides to bind to GRP94 [35,36,37]. Like the cytoplasmic isoforms of HSP90, GRP94 has the NTD, MD, CTD, and charged linker domains. However, there are some differences between the domains of these isoforms. A major difference between the isoforms is the length and sequence of the N-terminal amino acid sequence [38]. Another difference is that the charged linker region of GRP94 is shorter, rich in lysine residues, more acidic, and contains many calcium-binding sites [14].

TRAP1 (tumor necrosis factor receptor-associated protein 1) is another isoform of HSP90, present in the mitochondria. It is mainly present in the mitochondrial matrix but is also present in the intermembrane space to a lesser degree [39,40]. It shares high homology with cytoplasmic HSP90, and like the cytoplasmic HSP90, it consists of the three main domains, which are the NTD, MD, and CTD domains [2,41]. However, it does not have the charged linker domain. It also lacks the C-terminal MEEVD motif [42]. TRAP1 has 10 times more binding affinity to ATP, and its expression can be increased by up to 200 times more by heat shock [40,43,44].

## 3. Interactions with Co-Chaperones

Co-chaperons are proteins that aid chaperone proteins. They assist HSP90 throughout its conformational cycling necessary for normal function, help recognize substrates, aid the polypeptide in translocation, and supply additional enzymatic activity [13]. Most co-chaperones enable the recruitment of other substrate proteins. However, some of these co-chaperones—such as the client isomerases, phosphatases, and ligases—also provide supplementary enzymatic activity to the chaperone complex [45,46,47].

Tetratricopeptide repeat (TPR) domain-containing proteins are the predominant class of co-chaperones, and they bind to the MEEVD motif in the C-terminus of HSP90 [48,49,50,51]. Some co-chaperones that contain a TPR domain are C-terminus of HSP70-interacting protein, Hop, cyclophilin 40, FK506-binding protein, and protein phosphatase 5 (PP5) [13].

In addition to the TPR containing co-chaperones mentioned, there are also non-TPR containing co-chaperones. An example is Aha 1 (activator of HSP90 ATPase homolog 1), a co-chaperone that increases ATPase activity, thereby improving the function of HSP90 [28,52]. Cell division cycle 37 (Cdc37) is another such co-chaperone, and it is mostly linked to the formation of tumors due to its association with mutant kinases that drive cancer progression [53]. P23 is another co-chaperone, and its function is complexing nuclear hormone receptors and HSP90. The interactions of p23 are not as limited as Cdc37, as indicated by the broad range of HSP90 client complexes that p23 was found in [54,55,56,57].

While Aha1 increases ATPase activity, the co-chaperones Cdc37, p23, and Hop inhibit ATPase activity, thereby regulating HSP90 [13].

Most of the co-chaperones mentioned above are co-chaperones of cytoplasmic HSP90α and HSP90β. Unlike HSP90α and HSP90β co-chaperones, the interactions of isoforms GRP94 and TRAP1 co-chaperones are only partially understood due to their location within the endoplasmic reticulum and the mitochondria, respectively [42,58].

## 4. HSP90 Chaperone Cycle

As stated above, HSP90 is a dimer. It alternates between an open state and a closed state. When it is in the open conformation, the two N-terminals are separate and free to bind client proteins [59]. The N-terminals are brought together following the binding of ATP, which prompts the ATP pocket lid to close. These events lead to the dimerization of HSP90 into a compacted and closed conformation [59,60]. When in the closed state, the chaperone confines client proteins within it [61]. The binding of ATP and the ATPase activity of HSP90 are what propel the chaperone cycle [60].

**Figure 2:**
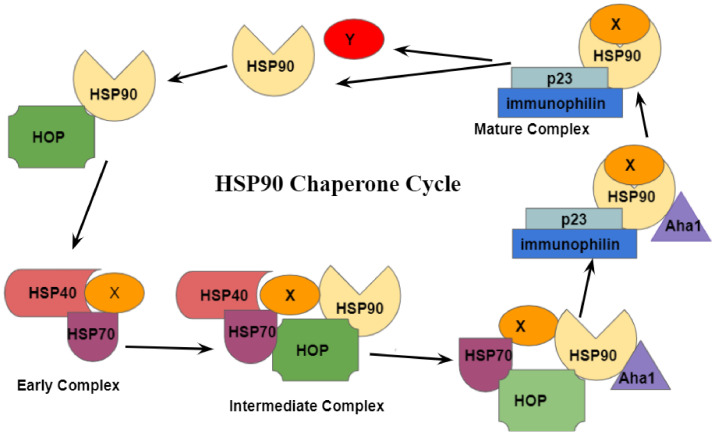
HSP90 chaperone cycle depicting interactions of co-chaperones and client proteins with HSP90.

As explained above, HSP90 is conserved across all organisms. Studies in steroid receptor maturation on yeast HSP90 have been extensively conducted to understand the HSP90 chaperone cycle [62]. As depicted in Figure 2, the HSP90 chaperone cycle occurs along with many of the co-chaperones mentioned in the previous section.

**Figure 3:**
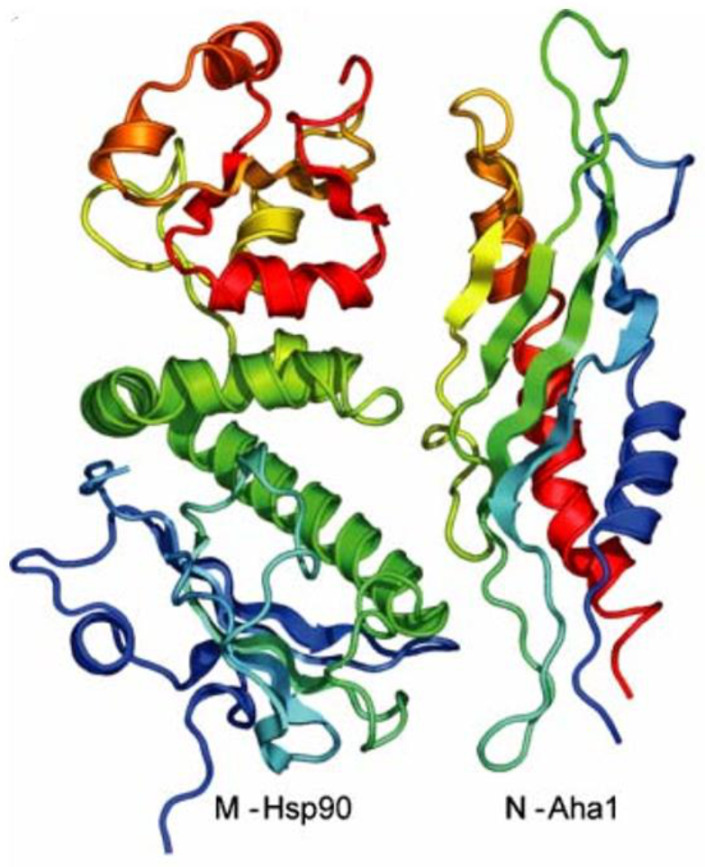
Crystal structure of the middle domain of HSP90 interacting with the N-terminal domain of Aha1 [65].

The cycle begins with the binding of a newly synthesized or misfolded steroid receptor to the HSP70/HSP40 complex. The complex comprising the steroid receptor and the HSP70/HSP40 complex is known as the early complex. The HSP70/HSP40 complex then associates with HSP90, which is in the open conformation. This association yields the intermediate complex. The association between the HSP70/HSP40 complex and the open HSP90 molecule is possible due to Hop, a co-chaperone that simultaneously interacts with both HSP90 and HSP70 [8]. Hop allows for the transfer of client proteins from HSP70 to HSP90. It does so by binding its middle TPR domain to both the N-terminal domain and the MEEVD motif of the C-terminal domain of HSP90 [1]. In doing so, Hop prevents interactions in the N-terminal domain obstructing ATPase activity [61,63,64]. As depicted in Figure 3, the N-terminal domain of Aha1 interacts with the three subdomains of the elongated middle section of HSP90 [65].

**Figure 4:**
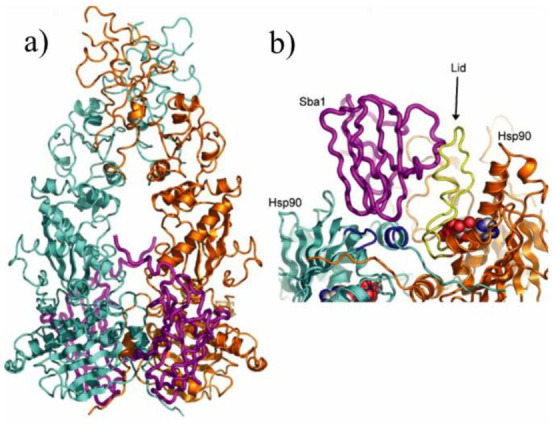
(**a**) Crystal structure of HSP90 in complex with p23/Sba1. Dimers of HSP90 are individually colored teal and orange, and p23/Sba1 is depicted in violet [65]. (**b**) Closeup image of p23/Sba1 interacting with the ATP-bound, closed-lid conformation of HSP90 [65].

This interaction drives conformational modifications that allow for ATP binding [66]. Following the binding of ATP to HSP90, Hop is replaced by p23 and immunophilins. As shown in Figure 4, p23 interacts only with an N-terminal domain of the HSP90 dimer that has already bonded with ATP in preparation for ATP hydrolysis.

Furthermore, circular dichroism spectroscopy and isothermal titration calorimetry both suggest that p23 binds to HSP90 in a 1:2 stoichiometry, and the binding of one p23 to an HSP90 active site results in a decreased likelihood of a second p23 binding [65]. At this stage, the intermediate chaperone complex has been converted to the mature chaperone complex [66]. ATPase activity in HSP90 is then driven by Aha1 and immunophilins [28,67,68]. Amid these processes, the client protein is altered into the correct form. Following the hydrolysis of ATP, the correctly folded client protein is released, and HSP90 returns to its open state [61]. Recent studies show that the mechanisms used in the yeast HSP90 chaperone cycle are also conserved in humans. However, the turnover rate of the human HSP90 chaperone cycle is slower than it is in yeast [59].

## 5. Differences between HSP90 in Normal Cells and Tumor Cells

The three major distinguishing mechanisms that differ between normal and tumor cells are the induction of HSP90 mRNA and proteins, protein activation by client association or post-translational modifications, and the localization of HSP90 to ectopic cellular compartments [13]. These differences make HSP90 a target protein in cancer therapy, despite it being a common housekeeping protein that is abundantly present in normal cells.

## 6. Induction of HSP90 mRNA and Protein

As stated in the introduction, HSP90 is present in abundance during normal cell conditions, specifically making up about 1–3% of total cellular protein [69,70,71,72]. However, in response to stress, such as heat, nutrient deficiency, and oxygen deficiency, which are commonly associated with tumor cells, heat-shock factor 1 (HSF1) is released from tight regulation at the post-translational level and forms a trimer, which relocates to the nucleus and induces increased HSP90 expression [13]. This upregulation of HSP90 is said to be what enables tumor cells to survive the harsh microenvironments by allowing for the persistence of mutations that spur malignancy [10].

For example, in hormone and protein kinase-dependent breast cancer, the level of HSP90 expression has correlated well with the survival outcome of patients and is a determining factor in survival outcome [13]. Compared with normal noncancer tissue, cancer tissue has exhibited greater levels of HSP90 expression [73]. Moreover, a study conducted at Yale University by Pick et al. on the association between HSP90 expression and breast cancer showed that a high expression level of HSP90 is associated with the risk of more malignant cancer that is less responsive to treatment [74]. Furthermore, after evaluating HSP90 gene expression from profiles of over 4000 breast cancer patients from 23 databases, annotated with overall survival data from over 1000 patients, biostatisticians confirmed a link between poor overall survival and high levels of HSP90 expression [75].

Although high expression of HSP90 is associated with tumor growth and is believed to be an oncogenic signaling node for cancer malignancy, some normal tissues, such as the bladder, spleen, and brain, exhibit a higher expression of HSP90 when the ratio of HSP90 to total protein is observed [72,76]. Because elevated levels of HSP90 are not resulting in tumorigenesis and cancer malignancy in these normal tissues, there may be a difference between HSP90 protein in tumor cells and HSP90 found in normal tissue. The fact that HSP90 has more than 30 sites where post-translational modifications can take place may explain some differences between HSP90 found in normal cells and those found in tumor cells [13].

## 7. Protein Activation

HSP90 can perform its functions due to its interactions with client proteins [77,78,79,80]. More than 400 discovered client proteins facilitate signal transduction pathways that dictate cell growth, apoptotic evasion, differentiation, and metastasis. Post-translational modifications influence the association of HSP90 with client proteins. Client association has become a means of distinguishing tumorigenic HSP90 from normal HSP90 [13].

Oncologists can use HSP90 as a drug target for cancer treatments because of the high affinity to HSP90 inhibitors that HSP90 in tumor cells exhibit. Normal HSP90 carrying out normal functions and making up most total cellular HSP90 is not inhibited by the same dose of inhibitor drugs that inhibit HSP90 in tumor cells. Therefore, the following can be hypothesized: HSP90 has low-affinity interactions with client proteins, which are regulated by low-affinity binding and the release of ATP and ADP, but upon mutation or deregulation, which is present in the cancer phenotype, many of these client proteins may display atypically stable interactions with HSP90, representing the active state that has a higher affinity for inhibitor drugs [13].

In a 2003 *Nature* paper, Kamal et al. stated that tumor cell HSP90 exists completely in multichaperone complexes. These complexes were said to increase ATPase activity in HSP90 and increase the affinity of HSP90 to the inhibitor 17-AAG by a 100-fold. As far as co-chaperone involvement, Kamal et al. showed that the highest ATPase activity occurred when HSP90 was associated with the co-chaperones HSP70, HSP40, Hop, and p23 [81]. Based on their study results, Moulick et al. proposed that HSP90 in tumor cells form biologically distinct complexes. The majority of cancer cell HSP90 is functionally similar to the HSP90 of normal cells; however, a distinct fraction of HSP90 that is functionally different and interacts with the oncogenic proteins required to maintain tumor cell survival also exists. This distinct fraction provides specific responses to the specific stress factors that the tumor cell undergoes. Moulick et al. also found that a statistically significant difference did not exist between the total HSP90 level present in mouse tumors and noncorresponding normal tissue [69]. However, the ATPase activity of HSP90 in the tumors was higher and so was their affinity for HSP90 inhibitors, which supports that transformation and malignancy cannot be explained solely by the elevated expression of HSP90 [81].

Unfortunately, attempts to replicate the work of Moulick et al. have failed to demonstrate the specific complex of HSP90 present in cancer cells. Affinity-based approaches might lead to client misidentification, but approaches examining the client proteins’ fate in an HSP90 inhibitor’s presence may be more effective. Defining a generally activated state of HSP90 in cancer remains difficult. Nonetheless, progress is being made in distinguishing between cancer HSP90 and normal tissue HSP90 based on the post-translational modifications. These post-translational modifications influence ATPase activity and the localization of HSP90, affecting the association of HSP90 with other proteins [13].

## 8. Ectopic Localization

Post-translational modifications influence the localization of HSP90, which has demonstrated its significance in that ectopic localization can contribute to the progression of a more malignant case of most cancers. HSP90 is no longer thought of as being located entirely within the cell. It is present on the plasma membrane of various cancer cells and is also secreted into the extracellular space. Cell surface HSP90 is generally present in higher levels on the cancer cells’ surfaces than on the normal cells’ surfaces. This makes cell surface HSP90 an attractive drug target against metastatic pathways dependent on aid from HSP90 for the invasion and migration into the cell [13].

Like intracellular proteins, extracellular proteins require chaperoning. Extracellular HSP90 is thought to have a chaperoning function similar to intracellular HSP90 [13]. It is thought that extracellular HSP90a functions with the co-chaperones Hsp70, Hsp40, Hip, Hop, and p23 to assist in cleaving and activating MMP-2. Since only low ATP levels are present in the extracellular environment, extracellular HSP90 can function independently of ATP [82]. Normal cells secrete HSP90 in response to stress, but cancer cells secrete HSP90 consecutively [83].

Plasma concentrations of HSP90 and tumor malignancy in clinical cancer patients were shown to have a positive correlation [84]. Thus, the effects of inhibiting extracellular HSP90 can allow for progress in effective cancer treatment. Many researchers have also already shown that blocking or neutralizing secreted HSP90 inhibits the metastasis of cancer [85,86]. Although a good understanding of extracellular HSP90 expression mechanisms has not been established, specific environmental stresses and growth factors have been linked to the stimulation of secretory pathways [83,87]. As stated above, post-translational modifications influence the localization of HSP90. Specifically, post-translational modifications, such as acetylation and phosphorylation, influence the secretion of HSP90 [88,89].

Although the specific mechanisms that enable HSP90 secretion are not yet clearly understood, it is well understood that HSP90 plays significant roles in the development of a nonmotile tumor cell into a motile and invasive cell [13]. Specifically, extracellular HSP90 was significantly involved in mediating cell invasion since it interacts with and activates matrix metalloproteinase-2 (MMP2) [83]. Tumor invasion occurs in three steps: the degradation of the extracellular matrix, adhesion, and migration of the cancer cells. HSP90 plays a role in each of these steps, most likely linked with chaperoning functions within the cell that allow for increased malignancy [13,90].

## 9. Contribution of HSP90 to Malignant Behavior

The metastasis of tumor cells characterizes a malignant behavior in cancer. The secretion of HSP90α plays a role in enhancing the tumor cells’ invasiveness, which is a necessary first step in metastasis. Enhanced invasiveness occurs when HSP90α is secreted via exosomes from invasive cancer cells and activates matrix metalloproteinase-2 (MMP-2) [91].

A better understanding of HSP90α and its secretion pathway is necessary to analyze the contribution of HSP90 to malignancy in cancer.

McCready et al., researchers from Tufts University and Protech Laboratory, verified that two HSP90α isoforms existed in MDA-231 breast cancer cells and determined the relative amounts of the two isoforms. The major isoform was the classical ten-exon isoform (AA1-1), while the other isoform, which was present in only minimal amounts, was AA1-2, with two additional exons. McCready et al. also verified that HSP90α is not secreted by the classical secretory pathway or in an isoform-specific manner but rather via exosomes [91]. Regarding the contribution of HSP90α to cancer malignancy, recent reports suggest that the release of exosomes containing HSP90α by invasive cancer cells could result in the increased motility of tumor cells. In a test conducted by McCready et al., the MDA-231 cells treated with the control variable had a normal morphology as opposed to the cells exposed to exosomes for 16 hours, which displayed a more polarized shape associated with a motile phenotype exhibiting a more linear cell shape and a large cell surface area [91]. To verify if the effect observed was partly due to the HSP90α present in the exosomes, the cells were exposed to the recombinant HSP90α protein. HSP90α treated cells became more polarized than control-treated cells but less polarized than the cells exposed to exosomes. Furthermore, to determine if cell morphology changes result in increased cell motility, McCready et al. performed wound-healing assays using SUM159 breast cancer cells and A172 glioma cells, which are highly invasive and motile cells better suited to the wound-healing assay. The different treatments were recombinant HSP90α, exosomes, and exosomes plus a function inhibiting HSP90α antibody. For both cell types, cells treated with either the recombinant HSP90α or with exosomes were significantly more motile than cells treated with the control [91].

As stated above, the role of HSP90α in activating MMP-2 is what enables it to enhance the invasiveness of tumor cells. MMPs are vital for cancer cell invasion because of their role in the digestion of the extracellular matrix components, allowing cancer cells to invade the bloodstream [92]. MMPs also facilitate the migration of cancer cells. MMP-2, specifically, can cleave the adhesive contacts and the cellular networks that are important for adherence of cells to the basement membrane, thereby enabling the migration of cancer cells [93,94,95]. HSP90α forms a complex with co-chaperones HSP70, Hop, HSP40, and p23, in addition to the client protein MMP-2, both within the cell and within an extracellular medium [96,97]. HSP90α is reliant on co-chaperones for the recruitment of client proteins; thus, the interaction between HSP90α and its client proteins is increased in the presence of these co-chaperones [96,98]. In this case, the co-chaperones HSP70, Hop, HSP40, and p23 enhance the interaction between HSP90α and MMP-2. McCready et al. were able to verify this by performing co-immunoprecipitations with HSP90α and MMP-2, either alone or in the presence of the co-chaperones HSP70, Hop, HSP40, and p23 [82]. The involvement of HSP90α in the activation of MMP-2 was supported by the significantly decreased levels of activated MMP-2 that resulted when HSP90α was removed from the complex [82].

The following description is the process of HSP90α activation of MMP-2: HSP90α interacts with the C-terminal hemopexin domain of MMP-2. This interaction protects MMP-2 from inactivation processing [99]. Almost all MMPs have two terminal globular domains (catalytic and hemopexin domains) connected by an unstructured linker. Recent studies have determined the linker to be flexible, allowing for the conformational change of MMPs from a compact into an elongated structure [100,101,102]. When MMP-2 undergoes a conformational change from a compact to an elongated structure, its linker domain becomes loose and is exposed. This may then provide access for cleavage by degrading enzymes. In the presence of HSP90α, which binds with MMP-2 via the hemopexin domain, the elongated structure would be stabilized, and the cleavage site would be protected [99].

In addition to MMP-2, HSP90α also activates other extracellular proteins. One of these proteins is the tissue plasminogen activator protein (tPA). Annexin II, a protein secreted via exosomes, associates with HSP90α and binds to tissue plasminogen activator protein and plasminogen [103]. This binding begins the process of converting plasminogen to plasmin, a protease [104]. Plasmin is important to cancer malignancy since it can play a critical role during multiple steps of cancer invasion and metastasis. It does so by inducing the degradation of various extracellular matrix proteins and activating certain growth factors leading to aggressive cancers [105]. Moreover, the results of the tests performed by McCready et al. indicate that the activation of plasmin by HSP90α participates in stimulating cell motility [81].

## 10. HSP90 Inhibitors

**Figure 5:**
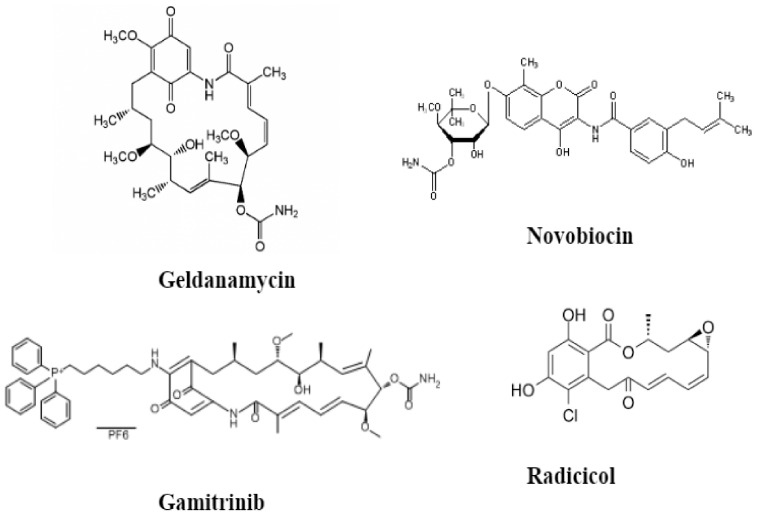
The chemical structures of four major natural HSP90 inhibitors.

HSP90’s critical role in cancer progression and the differences between normal HSP90 and cancer-related HSP90 are well understood. Thus, HSP90 has become a drug target. Shown in Figure 5 are HSP90 inhibitors have been thoroughly researched in terms of the domains the inhibitors bind to and which groups of the inhibitors are involved [106].

Geldanamycin (GA), the first-discovered HSP90 inhibitor, is a naturally occurring benzoquinone ansamycin antibiotic [107]. It binds competitively, directly to the ATP binding site in the N-domain of HSP90. In doing so, it blocks nucleotides from binding to HSP90, confining HSP90 in its ADP-bound conformation at the intermediate complex of the chaperone cycle [108]. This prevents the conformational change of HSP90, rendering HSP90 unable to clamp around a client protein [109,110,111,112]. The client protein cannot bind to HSP90 and undergoes ubiquitination and proteasomal degradation [109,113]. Despite having exhibited potent effects against cancer activities, GA’s high hepatotoxicity and inadequate solubility make it unable to be used clinically as a drug candidate [114,115]. The different derivatives with the same potent anticancer effects but with better toxicological properties were thus synthesized. An example of a GA derivative is 17-AAG, which is more hydrophilic and has been used with success in preclinical and clinical studies [116].

Radicicol (also known as monorden) is another natural product inhibitor of HSP90. It is a 14-membered macrolide, initially isolated from the fungus *Monosporium bonorden*. Like GA, radicicol acts as a nucleotide-mimicking compound and competes for the N-terminal ATP binding pocket of HSP90. Radicicol has a much higher affinity to this pocket than ATP [117]. As observed in the interactions between GA and HSP90, the interaction with radicicol restrains HSP90 in its ADP-bound conformation, resulting in the destabilization of client proteins [111]. Radicicol has displayed potent antiproliferation effects in vitro; however, it has not displayed anticancer activity in vivo, which may be because of its low biological stability [118,119]. Following this discovery, radicicol derivatives with better biological stability and in vivo efficacy have been synthesized [120].

Many new inhibitors have been discovered, and many derivations of geldanamycin and radicicol have been synthesized [107]. An example is radamide, which was designed based on the co-crystallization structures of the GA/HSP90 N-domain and the radicicol/HSP90 N-domain [112,121]. This chimeric compound contains aspects of both radicicol and geldanamycin. Specifically, it has the resorcinol ring of radicicol and quinine ring of geldanamycin [110]. In breast cancer cells, radanamycinamide has displayed potent inhibition effects of HSP90 in a low micromolar range [122]. Another inhibitor is PU3. It is purine based and structurally resembles ATP, allowing it to bind to the N-terminal domain of HSP90 and inhibit the growth of breast cancer cells [121,123].

Another HSP90 inhibitor is novobiocin. It is a coumarin antibiotic isolated from Streptomyces species [124,125]. Unlike GA and radicicol, which target the N-terminal domain, this inhibitor targets the C-terminal domain of HSP90 and binds to the C-terminal ATP binding site [126]. However, similar to the HSP90 inhibition by N-terminal inhibitors, HSP90 inhibition by novobiocin resulted in the destabilization of various HSP90 client proteins such as Her-2, Raf-1, and p53 mutant via the ubiquitin-proteasome pathway [124,127,128]. The C-terminal and N-terminal domains of HSP90 may be involved in an allosteric regulation in which ligand interaction with one site may be influenced by ligand occupancy of the other site [124,125,129]. Clorobiocin and coumermycin A1 are related coumarin antibiotics that bind to the C-terminus of HSP90. In comparison to novobiocin, these antibiotics have displayed improved activity [124,130].

As discussed earlier, mitochondrial HSP90, known as TRAP1, is also present within the cell. Its role in tumorigenesis is in the prevention of the initiation of induced apoptosis. Gamitrinib is a resorcinolic inhibitor that specifically targets and acts on mitochondrial HSP90. Its inhibition of mitochondrial HSP90 induces a sudden loss of membrane potential, causing membrane rupture and apoptosis initiation. Gamitrinib is highly selective and does not affect normal cells [131].

As of now, there are more than ten different HSP90 inhibitors that are in various stages of clinical development. This includes inhibitors such as IPI504, NVP-AUY922, and STA-9090 [132]. So far, the results from these clinical developments appear to ensure progress in cancer treatment, yet there are still many unanswered questions [107].

## 11. Use of HSP90 Inhibitor in Different Cancer Types

17-AAG, a derivative of the HSP90 inhibitor geldanamycin, entered Phase I trials in 1999 and has completed Phase I testing [133,134]. Positive effects were observed in its use for treating melanoma, breast cancer, prostate cancer, and multiple myeloma [134,135,136,137,138]. The Phase II trials focused on tumor types with specific HSP90 chaperoning targets. Examples of these tumor types are leukemia-expressing Bcr-Abl and Her-2 positive breast cancer [134,139].

IPI-504 is a water-soluble hydroquinone hydrochloride analog of 17-AAG [140]. It is in Phase I and Phase II clinical trials to determine its capability in the treatment of cancer that has developed resistance to tyrosine kinase inhibitors, such as Philadelphia chromosome-positive chronic myelogenous leukemia (CML) [106].

Ganetespib is a resorcinol-containing triazolone agent. It has undergone Phase I and Phase II studies for solid tumors as well as hematological malignancies. It has also undergone Phase III clinical trials in combination therapy with docetaxel to treat patients with advanced NSCLC (non-small cell lung cancer) [141]. A 50% response rate was observed in heavily pretreated NSCLC patients with tumors harboring ALK rearrangement when the inhibitor was used alone [132]. A 67% response rate has been observed in a Phase I trial evaluating combination therapy consisting of ganetespib and crizotinib in metastatic NSCLC patients with ALK-rearranged tumors that were not previously treated with crizotinib [141]. Ganetespib has also shown success in treating other types of cancers. For example, recent studies have shown that ganetespib effectively decreased tumor growth in a dose-dependent manner in eight cell lines originating from papillary, follicular, anaplastic, and medullary thyroid cancers [142].

Luminespib is a nongeldanamycin, isoxazole resorcinol derivative HSP90 inhibitor. It has displayed potent efficacy in the treatment of diverse types of tumors [143]. Response rates ranging from 10–25% have been observed in Phase II studies in EGFR-mutated and ALK-rearranged NSCLC and HER2+ breast cancer resistant to standard treatment [144,145]. Early-state antitumor activity has also been seen in NSCLC patients with EGFR exon 20 insertions, a rare subtype causing resistance to treatment with EGFR-specific tyrosine kinase inhibitors [146] Currently, luminespib is undergoing Phase II testing in advanced ALK-positive NSCLC [141].

## 12. Application in Modern Cancer Therapy

When HSP90 inhibitors are administered to animals with human tumors, the tumors stop growing, yet the tumors often begin growing again when the inhibitor is withdrawn. This also occurs in patients that have solid tumors and are being treated with various HSP90 inhibitors. This behavior suggests that HSP90 inhibitors may have limited use as a monotherapy. However, recent studies have shown that certain types of tumors may respond more sensitively to HSP90 inhibition than other types. Recent observations suggest that monotherapy with HSP90 inhibitors may be suffice for facilitating cellular death and reduce tumors [13].

Recent Phase II studies with only ganetespib, a synthetic HSP90 inhibitor, have yielded a response rate of 50% in non-small cell lung carcinoma patients whose tumors contained ALK translocations [147]. Although monotherapy has shown remarkable success in some studies, combination therapy involving the use of HSP90 inhibitors with existing chemotherapeutics or successful drugs such as Herceptin, lapatinib, or Gleevec has shown better success and has become a topic of interest. For example, a combination of SNX5422, an HSP90 inhibitor, and Herceptin was synergistic and resulted in dramatic and persistent tumor reduction. These effects lasted even after this combination was withdrawn [13].

Heterogeneity within individual tumors can facilitate tumor evolution and adaptation, impeding personalized treatment methods that rely on single tumor biopsy sample results [148]. Oncogene switching plays a significant role in the development of cancer drug resistance. HSP90 inhibitors can help prevent drug resistance in tumors since oncogenes are highly dependent on HSP90 to chaperone their unstable conformation caused by mutations. This heavy reliance can also be called an oncogene addiction. Because several oncoproteins are dependent on HSP90 activity, an HSP90 inhibitor can affect multiple targets and pathways, preventing oncogene switching [149,150]. By preventing oncogene switching, HSP90 inhibitors can reduce or combat drug resistance, yet cancer resistance to HSP90 inhibitors could also occur since cancers evolve and adapt [13]. However, silencing co-chaperones, such as p23, Aha1, or Cdc37, has resulted in cancer cells becoming dramatically sensitized to HSP90 inhibition [151,152,153].

As the interactions of HSP90 with other heat-shock proteins and co-chaperones are understood, combination therapies involving HSP90 inhibitors and inhibitors of other heat-shock proteins has become a topic of interest, specifically the association of HSP90 with HSF1 and HSP70. Although shown to be beneficial overall, the inhibition of HSP90 has some negative effects. One of these effects is the trimerization and activation of HSF1 [154,155]. Since increased levels of HSF1 are associated with oncogenesis, ways to minimize its negative effects are important for use alongside HSP90 inhibitors. Elevated HSF1 also causes the upregulation of HSP70, of which the inducible form can facilitate oncogenesis in tumors. Targeting HSP70 has been observed to be valuable since the inducible form of HSP70 is overexpressed by tumors much more than the constitutive form [156]. Thus, combination therapy involving both HSP90 inhibitors and HSP70 inhibitors could bring about greater success in cancer treatment by eliminating the negative effects of inducible HSP70 brought about by HSP90 inhibition [13].

## 13. Conclusions

This review attempts to summarize current research on the role of HSP90 in cancer and HSP90 inhibitors as an effective form of cancer therapy. In addition, the role of post-translational modifications (PTM) in HSP90 expression and the interaction of HSP90 with proteins related to hallmarks of cancer is discussed. Progress in understanding the role of HSP90 overexpression in cancer progression and the differences between HSP90 in normal cells and HSP90 in cancerous cells has made HSP90 a target chaperone protein for a cancer therapy drug. Natural HSP90 inhibitors have been discovered, and their properties have led academics to synthesize derivatives with improved efficacy and lower toxicology. The use of HSP90 inhibitors as monotherapy has brought about some successes, as well as instances in which tumor reduction only occurred when the HSP90 inhibitors were administered, returning after the inhibitors were withdrawn. Moreover, combination therapies involving HSP90 inhibitors and other cancer drugs have shown high efficacy, and certain combination therapies have allowed for significant tumor reductions even after the therapy’s withdrawal. Though overwhelmingly positive, HSP90 inhibition also has negative effects associated with tumorigenesis, such as elevated levels of HSF1, leading to the overexpression of HSP70. To negate these effects, researchers are looking into combination therapies involving HSP90 inhibitors and HSP70 inhibitors. A major obstacle in current cancer research is the potential resistance to inhibitors, but the combined use of HSP90 inhibitors with other drugs can help combat drug resistance.

Studies in the role of HSP90 in cancer have gone from the first Phase I trials of 17-AAG to the discovery and synthetization of HSP90 inhibitors, such as ganetespib and luminespib, that are being researched today. Despite substantial advancement, HSP90 inhibitors have not yet been approved for clinical use because the efficacy to curb cancer progression is not up to the expected levels. Additional clinical trials have the potential to more accurately gauge the effectiveness of HSP90 inhibitor derivatives on various cancer types, which allows for a more effective and targeted approach to cancer therapy in the times to come.
